# Magnetic Resonance Imaging Angiography of Physiological and Pathological Pregnancy Placentas Ex Vivo: Protocol for a Prospective Pilot Study

**DOI:** 10.2196/35051

**Published:** 2022-08-10

**Authors:** Matthieu Dap, Bailiang Chen, Claire Banasiak, Gabriela Hossu, Olivier Morel, Marine Beaumont, Charline Bertholdt

**Affiliations:** 1 Obstetric and Fetal Medicine Unit Centre Hospitalier Régional Universitaire of Nancy Nancy France; 2 Department of Foetopathology and Placental Pathology Centre Hospitalier Régional Universitaire of Nancy Nancy France; 3 INSERM U1254 IADI Vandoeuvre-lès-Nancy France; 4 INSERM CIC-IT 1433 Innovative Technology University of Lorraine and University Hospital of Nancy Nancy France

**Keywords:** MRI, magnetic resonance imaging, placenta, IUGR, intrauterine growth restriction, preeclampsia, PE, uterine, intrauterine, pregnancy, vasculogenesis, pathology, physiology

## Abstract

**Background:**

Preeclampsia (PE) and intrauterine growth restriction (IUGR) are 2 major pregnancy complications due to abnormal placental vasculogenesis. Data on whole fetoplacental vasculature are still missing; hence, these pathologies are not well understood. Ex vivo magnetic resonance imaging (MRI) angiography has been developed to characterize the human placental vasculature by injecting a contrast agent within the umbilical cord.

**Objective:**

The primary objective of this study is to compare the placental vascular architecture between normal and pathological pregnancies. This study’s secondary objectives are to (1) compare texture features on MRI between groups (normal and pathological), (2) quantitatively compare the vascular architecture between both pathological groups (pathological IUGR, and pathological PE), (3) evaluate the quality of the histological examination in injected placentas, and (4) compare vascularization indices to histological characteristics.

**Methods:**

This is a prospective controlled study. We expect to include 100 placentas: 40 from normal pregnancies and 60 from pathological pregnancies (30 for IUGR and 30 for PE). Ex vivo MR image acquisition will be performed shortly after delivery and with preparation by injection of a contrast agent in the umbilical cord. The vascular architecture will be quantitatively described by vascularization indices measured from ex vivo MRI angiography data. Comparisons of vascularization indices and texture features in accordance with the group and within comparable gestational age will be also performed. After MR image acquisition, placental histopathological analysis will be performed.

**Results:**

The enrollment of women began in November 2019. In view of the recruitment capacity of our institution and the availability of the MRI, recruitment should be completed by March 2022. As of November 2021, we enrolled 70% of the intended study population.

**Conclusions:**

This study protocol aims to provide information about the fetal side of placental vascular architecture in normal and pathological placenta through MRI.

**Trial Registration:**

Clinicaltrials.gov NCT04389099; https://clinicaltrials.gov/ct2/show/NCT04389099

**International Registered Report Identifier (IRRID):**

DERR1-10.2196/35051

## Introduction

Placental dysfunction is a major cause of intrauterine growth restriction (IUGR) or preeclampsia (PE) and is responsible for maternofetal mortality and morbidity such as fetal death, preterm delivery, minor cognitive deficits, school problems, and metabolic syndrome in adulthood [1-3]. Normal placental development is an essential prerequisite for efficient placental function followed by fetal growth. Placental dysfunction is primarily due to a deficient remodeling of the spiral arteries in the myometrium during the first trimester [4]. These remodeling disorder leads to abnormal placental development with morphological and functional alteration of the placenta. The exploration of these alterations, functional and morphological, is important to not only understand the pathophysiology of IUGR and PE but also for screening or diagnostic techniques to improve antenatal care. Imaging tools are particularly suitable for this exploration.

As a consequence, some teams work on in vivo exploration of placental function using magnetic resonance imaging (MRI) or ultrasound imaging [5-7]. However, in vivo imaging has the disadvantage of having major difficulty in differentiating between the maternal and fetal sides, except for very advanced MRI techniques (DECIDE) for contrast-enhanced ultrasound imaging, which allows for the exploration of exclusively the maternal side and for MRI with gadolinium injection but is not allowed during pregnancy [8,9]. Besides, ex vivo studies seem to offer unique opportunities to explore placental physiology even outside the womb, where the placenta’s normal state is modified [10]. Two main tools have been described: ex vivo microcomputed tomography (micro-CT) and ex vivo MRI [11-13]. Regarding ex vivo micro-CT, Junaid et al [11] published a comparative analysis of placental vascularization between IUGR and normal placentas and showed a significant decrease in vessel length in IUGR placentas compared to normal ones.

Regarding ex vivo MRI, several studies described an MRI-compatible perfusion chamber allowing the exploration of dynamic placental perfusion during MRI scanning [14,15]. Ex vivo MRI could also be used to explore placental function.

Other studies have described a protocol for ex vivo angiography of the fetal vessels, including Link et al [16] and Chen et al [17]. The described technique allows for imaging of the architecture of the whole fresh human placental vascular tree by injection of oil in the umbilical cord vessels. This protocol can reveal up to the sixth level of placental vascularization at a submillimeter quasi-isotropic resolution [17]. Link et al [16] recently compared IUGR and normal placental vascular trees using ex vivo angiography [16]. The main limitation of this work is that the understanding of placental structure and vasculature at different gestational stages is limited [18,19].

The main objective of our study is to compare the vascular architecture of the placenta in normal and pathological pregnancies; for example, PE and intrauterine growth restriction. Our secondary objectives are to (1) compare texture features on MRI between groups (normal and pathological), (2) quantitatively compare the vascular architecture between both pathological groups (pathological IUGR and pathological PE), (3) evaluate the quality of the histological examination in injected placentas, and (4) compare vascularization indices to histological analysis.

## Methods

### Trial Design

**Figure 1:**
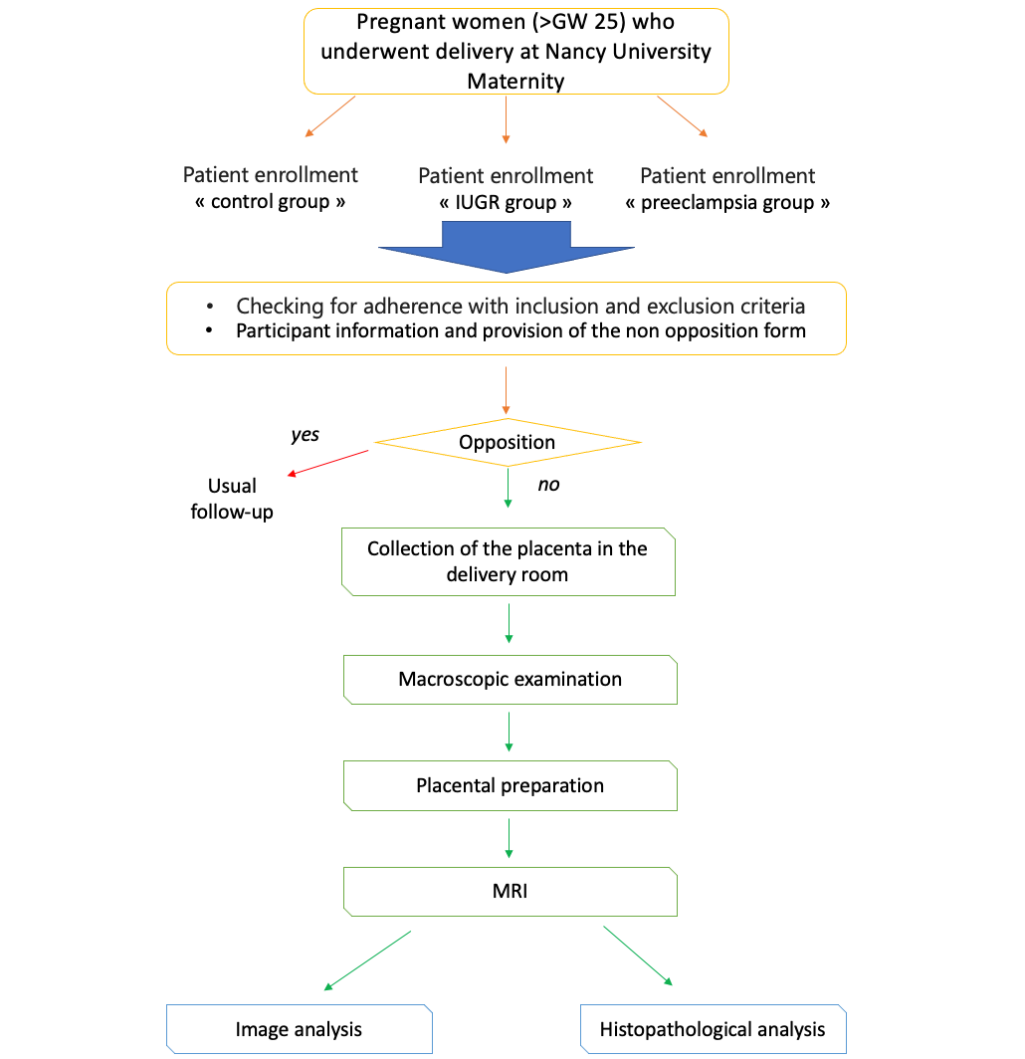
Study flowchart. GW: gestational week; IUGR: intrauterine growth restriction; MRI: magnetic resonance imaging.

The MRI Angiography of Physiological and Pathological Pregnancy Placentas *Ex vivo* (MAPLE) protocol is a prospective, monocentric (Maternité du Centre Hospitalier Régional Universitaire [CHRU] de Nancy, France), open, controlled, nonrandomized pilot study conducted at CHRU de Nancy. Figure 1 presents the study flowchart.

### Study Population

Enrolled women will be distributed into 3 groups (normal, IUGR, and PE) in accordance with their pregnancy status. The participants in this study are pregnant women in their third trimester in order for us to obtain comparable gestational ages between groups. Textbox 1 presents the inclusion and exclusion criteria. IUGR is defined as a birth weight less than the third percentile of the French growth curve (Association des Utilisateurs de Dossiers Informatisés en Pédiatrie, Obstétrique et Gynécologie, Lyon, France). PE is defined as a systolic blood pressure of ≥140 mm Hg and a diastolic blood pressure of ≥90 mm Hg associated with proteinuria of >300 mg per 24 hours after 20 weeks of gestation. Before inclusion, women will be informed of the aim, procedures, and predictable risks of the study (no identified clinical risk) by the investigator. Patients’ medical history, including smoking and obstetric history, will be collected. Medical information on the current pregnancy will also be collected (Textbox 2).

**Inclusion and exclusion criteria.**

**Inclusion criteria:**

- Already received complete information about the study and do not express any opposition to the use of their data
- Mandatory enrollment in a social security plan
- Gestational age of >25 weeks (and until at term)
- Having undergone either natural or Cesarean delivery and whose placenta is naturally completely separated
- For the normal group:
  Normal fetal weight (between the 10th and 95th percentile for age)
  No intrauterine growth restriction (IUGR) or preeclampsia (PE) suspected or confirmed
  No Doppler (fetal and umbilical) abnormality
- For the pathological group:
  Subgroup IUGR: IUGR defined by a fetal estimated weight of less than the third percentile of the antenatal curve, and confirmed in the postnatal stage; absence of associated PE
  Subgroup PE: clinical diagnosis of PE (high blood pressure associated with significative proteinuria); absence of associated IUGR

**Exclusion criteria:**

- Younger than 18 years
- Newborn with congenital disease (either suspected at birth or already diagnosed)
- Presence of only one umbilical artery
- Presence of a maternal pathology: gestational diabetes, pre-existing autoimmune diseases, or cancer
- Do not speak French or inability to understand the given information of the study
- Manual delivery
- Incomplete placenta
- Person referred to articles L.1126-6 and L-1126-8 of the public health code

**Data collected.**

**Habitus:**

- Smoking and addictions
- Drug consumption

**Obstetric data:**

- Gestational age, parity, and birth weight

**Clinical data:**

- BMI and age
- For preeclampsia: proteinuria, blood pressure (systolic and diastolic), and complications
- For intrauterine growth restriction: ultrasound data (biometry, umbilical artery Doppler, and estimated fetal weight)

**Magnetic resonance imaging data:**

- Morphological images of the placenta, vascularization indices, and texture indices

**Histological data:**

- Placental analysis (weight and morphological analysis)

### Placenta Preparation for Perfusion

**Figure 2:**
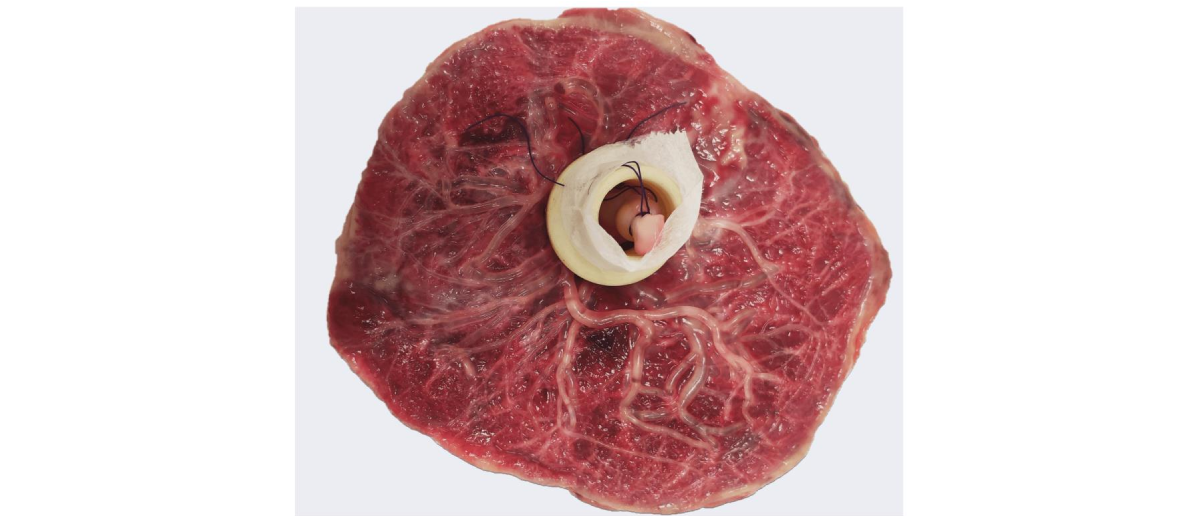
Placenta prepared for magnetic resonance imaging. The placental vascular tree is filled with oil. The sealed cord is placed within a plastic tube supporter designed and printed with a 3D printer.

All human placentas will be immediately collected and prepared for acquisition after natural vaginal delivery or Cesarean delivery to reduce the occurrence of blood clotting. The preparation procedure was based on Rasmussen’s procedure modified by Chen et al [17] to fit our requirements [20]. First, a gross macroscopic examination of the placenta will be performed as described by the Amsterdam Placental Workshop Group Consensus, including placental weight, dimensions, and descriptions of the umbilical cord, membranes, and lesions [21]. Next, fresh placentas will be placed in a water bath at 37 °C during preparation. The chorion and amnion will be trimmed and removed. The umbilical cord will be severed at a 5-cm length to its placental insertion point. Umbilical vessels will be catheterized. The placental vascular bed will be slowly rinsed with a water-based solution through catheters fixed to the artery. The wash will be stopped when the reflux from the vein catheter becomes completely clear. The contrast agent (B-oil for rotary vane pumps, Vacubraand) will be slowly injected through the umbilical arteries until outflow of the contrast agent in the vein is observed, to ensure microvessel filling. The arteries and veins need to be closed well at their entry points. The overall preparation time is 1 hour. The sealed cord will be placed within a plastic tube supporter (the residual umbilical cord part), which was dedicatedly designed and printed with a 3D printer (Figure 2). Within this holder, the umbilical cord was tightly attached and not compressed so that the primary level of the vein and artery vessel trees can be distinguished in the MR data for the subsequent vessel tree extraction algorithm, especially to be able to separate arteries and veins. After preparation, the placenta will be stored in a refrigerator at 4 °C.

### MR Image Acquisition

Prepared placentas will be taken out from the refrigerator and placed in the MR room for approximately 30 minutes before MR image acquisition in order to ensure a steady temperature during the whole acquisition procedure. It will be placed on top of a flat platform appropriate for placental size: one with a diameter of 22 cm, the other with a diameter of 22.5 cm. Both can be fitted inside a 20-channel head coil. Therefore, the placenta is well placed at the isocenter of the MRI bore so that the homogeneity of the magnetic field can be maximumly satisfied. The imaging protocol has been tuned and set up on a 3T Prisma Siemens MR scanner. A 3D Flash sequence is employed to acquire the data. The relevant parameters are 0.4 × 0.4 × 0.4 mm^3^, TR/TE 8/3.5 milliseconds, 512×512 acquisition matrix, bandwidth of 390 Hz, and 14 NEX. The field of view and number of slices can vary from placenta to placenta, but all will be adjusted to achieve the aforementioned acquisition parameters, especially the isotropic resolution. Neither parallel imaging nor partial Fourier acquisition strategies have been adopted. The mean overall acquisition time was 1 hour 40 minutes. After MR image acquisition, the placenta will be immediately sent back to the pathology laboratory for histological processing, being always maintained at 4 °C.

### Pathological Examination

After MR image acquisition, the entire placenta will be fixed in a 4% formaldehyde solution for approximately 1 month. Random samples of cord, membranes, and placental parenchyma will be submitted (4 blocks as a minimum and 1 block for each type of lesion) in accordance with the recommendations of the Amsterdam Consensus Statement and be subjected to histopathological evaluation [21]. Once the tissue block is embedded in paraffin wax, a 6-µm vertical section containing the entire thickness of the placenta will be taken and mounted for staining with hematoxylin and eosin.

Certified pathologists will read slides to establish a histopathological diagnosis. Gross and histological lesions of fetal and maternal vascular malperfusion will be reported. Fetal vascular malperfusion (FVM) will be classified as global/partial or segmental/complete, and high-grade FVM will be also reported [22,23]. Finding of maternal vascular malperfusion (MVM) we be classified into villous changes (infarcts, retroplacental hematoma, accelerated villous maturation, and distal villous hypoplasia) and vascular lesions (acute atherosis, persistent muscularization of basal plate arterioles, basal decidual vessel thrombosis, and mural hypertrophy of membrane arterioles) [22]. MVM will be graded as global/partial or segmental/complete [23].

### MR Image Analysis

All the placental data will be archived and managed by a local research image management system ArchiMed. A MATLAB program has already been developed to segment the acquired placenta data and extract the vessel tree structure [17]. Owing to the good contrast-to-noise ratio and signal-to-noise ratio of the MR images, a simple thresholding is sufficient to delineate the vessel tree. A fast-marching algorithm will be used to quantitatively characterize the placenta’s vessel tree. Separation of the artery and vein tree structure will be achieved with a homemade software written in C++ as described by Kerrien et al [24]; the segmented placenta vessel data will be rendered in 3D, and its skeleton will be overlapped with its 3D model using this software. Morphological parameters including total vessel density, artery and vein density (per cm^3^), and tortuosity and bifurcation density (per cm^3^) will be calculated automatically or semiautomatically. Total vessel density is defined as the overall vessel density regardless of whether the vessel is an artery or vein. It is calculated as the vessel voxel volume divided by the whole placenta volume, while artery and vein vessel density are defined as artery and vein volume divided by whole placenta volume, respectively. The 3-vessel density will be calculated automatically by the software. Calculation of tortuosity and bifurcation density are computed semiautomatically as the branching of the vessel network and bifurcations need to be checked by observers. Tortuosity is defined as the curvilinear distance and Euclidean distance between the starting and the end of a vessel path (in this case, a vessel branch) [24]. Bifurcation density is defined as the number of detected bifurcations divided by the placental volume. Radiomics features, as described by Vallières et al [25], will also be extracted to quantify the differences among the 3 groups of placentas [25]. The quantitative parameters will be described by their mean (SD), median, maximum and minimum, and n (%) values. Mean values between groups will be compared using the Student *t* test or Mann-Whitney test, unpaired in accordance with whether the data are normally distributed, depending the type of data. Linear regression models will be used to test the group and the effect of gestational age on the parameters considered.

### Outcomes

The primary outcome will be the vascular architecture, described by vascularization indices (total vascular density, arterial and venous density in cm^3^, tortuosity index, and branching index) measured from ex vivo MRI in both groups (physiology and pathology).

The secondary outcome measures are the following:

- Secondary objective 1: measurement of texture indices as established by Vallières et al [25].
- Secondary objective 2: measurement of vascularization indices and texture based on the participant group.
- Secondary objective 3: number of placentas with an appropriate histological quality to be examined microscopically.
- Secondary objective 4: statistical comparison between vascularization indices achieved through MRI and histological evaluation (presence or absence of FVM, presence of absence of MVM, if present: grading of the lesions).

### Follow-up

No specific follow-up has been planned for participants except for standard routine care. Any adverse events will be noted and reported.

### Patient and Public Involvement

Patients and the public were not involved.

### Sample Size Consideration

Given the exploratory nature of this study and in the absence of data allowing for sample size estimation, we consider it necessary to include 100 women: 40 women with a normal pregnancy and 60 women with a pathological pregnancy (30 women with an IUGR and 30 with PE).

In fact, to obtain a sufficiently precise characterization and to be able to use descriptive statistics applicable to large samples (mean, SD values), we will need to obtain at least 30 observations in each group.

### Data Collection and Management

An electronic case report file (e-CRF) will be created for each woman. The women's anonymity will be ensured by mentioning to the maximum extent possible their research code number, followed by the first letters of the last name and first name of the participant on all necessary documents or by deleting their names by appropriate means (whiteout) from the copies of source documents intended to document the study.

The MRI data will be anonymized and transferred via a secure server for storage and archiving directly in the ArchiMed database, reported to the CNIL (CNIL report number 1410005) because they cannot be transcribed in the e-CRF. The clinical data of the women are shown in Textbox 2.

### Statistical Analysis

Quantitative data will be described by their mean (SD), median, and maximum and minimum values. Qualitative data will be expressed as n (%) values. Between-group comparisons will be performed using the Student *t* test or the Mann-Whitney test, matched or unmatched, depending on the type of data. The chi-square test or the Fisher exact test will be used to compare the qualitative data between groups. The analyses will be performed with R software (R Foundation for Statistical Computing). No intermediary analysis is intended. The analyses will be performed on a per-protocol basis.

### Quality Control: Right of Access to Data and Source Documents

Medical data of each patient will only be transmitted to the responsible party (CHRU de Nancy) or any person duly authorized by the responsible party and, where applicable, to the authorized health authorities, under conditions guaranteeing their confidentiality.

The responsible party and the regulatory authorities may request direct access to the medical file for verification of the procedures and data of the clinical trial without violating confidentiality and within the limits permitted by laws and regulations.

For research purposes, processing of personal data of the study participants will be carried out. These data are collected and processed solely on the basis of the legal grounds provided for by statute and regulations in the context of the performance of the public interest missions of CHRU de Nancy, particularly those relating to ensuring and contributing to research and innovation (Article 6.1.e of the General Data Protection Regulation [GDPR]). The processing of personal data of the study participants is permitted by the exception provided for in Articles 9.2(i) and (j).

This data processing is part of the MR003 reference methodology that CHRU de Nancy has undertaken.

According to the GDPR, persons participating in research have the right of access to their data (Article 15), the right of rectification (Article 16), the right to erase their data (right to forget) under the conditions (Article 17), the right to limit the processing (Article 18), and the right to object to the processing of their personal data (Article 21). These rights are exercised with the Investigators, who will inform the research stakeholders as soon as possible.

The persons participating in the research also have a right to complain to the supervisory authority in France, namely, the Commission Nationale de l'Informatique et des Libertés (CNIL).

### Study Monitoring

Each patient's e-CRF must be consistent with the source documents.

### Data Management and Quality Control

Data management will be carried out by the clinical research team Centre D'investigation Clinical–Innovation Technological (CIC-IT) at CHRU de Nancy (INSERM CIC 1433). The MR imaging data will be automatically transferred to CIC-IT and be stored after verification in the ArchiMed database declared to the French authorities (CNIL declaration number: 1410005).

### Patient Data Protection

Each patient must be identified on the e-CRF and placental specimen with her initials and ID number indicating her order of inclusion in the study. The investigators must maintain a list of all the patients, including their ID numbers and full names.

Patients must be informed in writing about the possibility of audits by authorized stakeholders and regulatory authorities, in which case the relevant parts of study-related hospital records may be required.

Patients will also be informed that (1) the results obtained will be computerized and analyzed, (2) local laws will be applied, (3) their confidentiality will be preserved, and (4) they are entitled to obtain any information concerning the data stored and analyzed by the computerized system.

### Potential Risks Related to the Study

This study will comply at all times with the Good Clinical Practices defined by the Ministry of Health, France. In this study, only placental collection is intended to be performed. This study, therefore, does not expose any particular risk to the women.

### Ethical Considerations

The stakeholders and all investigators undertake to conduct this study in accordance with the Declaration of Helsinki (Ethical Principles for Medical Research Involving Human Subjects, Tokyo 2004) and its updates, the provisions of European Directive 2001/20-CE as transposed into French law by L. 2004-806 dated August 9, 2004, on public health policy and 2004-800 dated August 6, 2004, on bioethics and their implementation decrees, and to comply with the guidelines of Good Clinical Practices (ICH version 4 of May 1, 1996, and decision of November 24, 2006).

They undertake to adhere to all legislative and regulatory provisions that may concern the study. In accordance with Article L. 1123-6 of the Public Health Code, the responsible party has submitted the research protocol to the Nancy CHRU Ethics Committee (Comité d’éthique du CHRU de Nancy). The collection was declared to the Ministry of Education, Research and Innovation on October 3, 2019 (reference number DC-2019-3739). The study is registered under the number NCT04389099 on www.ClinicalTrials.gov.

The study will be conducted in accordance with the present protocol. The investigators undertake to respect the protocol in all aspects especially with regard to the information and delivery of an opposition form to each patient.

### Information Letter and Opposition Form

Research participants will be informed of the objectives and constraints of the study, their rights to refuse to participate in the study, or to withdraw from the study at any time. When all essential information has been conveyed to the subject and the investigators have ensured that the patient has understood the implications of participating in the trial, the placenta can be collected in the absence of patient opposition.

### Protocol Amendment

The responsible party must be informed of any proposed amendment to the protocol by the principal investigator. Changes, substantiative or not, must be described.

### Final Research Report

The principal investigator and the mandated biostatistician will collaboratively write the final research report. This report will be submitted to each of the investigators for review. Once consensus has been reached, the final version must be endorsed with the signature of each of the investigators and sent to the responsible party as early as possible after the effective end of the study. A report prepared in accordance with the reference plan of the competent authority must be forwarded to the CHRU Ethics Committee within a year after the end of the study. This final report is made available to regulatory authorities.

## Results

The enrollment of women began in November 2019. In view of the recruitment capacity of our institution and the availability of the MR images, recruitment should be completed by March 2022. As of November 2021, we enrolled 70% of the population.

## Discussion

This study aims to evaluate whole human placental vasculature using ex vivo MRI in a normal and pathological population. We hypothesize that the placental vascular tree is altered in case of IUGR or PE compared to normal pregnancy. Our study data will provide information about uteroplacental physiology and pathophysiology by the exploration of the fetal placental side.

To our knowledge, this is the first prospective, comparative, controlled study comparing the architecture of the placental vascular tree between normal and pathological pregnancies at the same gestational age.

The groups have been selected strictly on the basis of gestational age and neonate size at birth to assess the comparison. This technique has been previously evaluated in a pilot study carried out by our team; however, only the fetal side can be analyzed with this technique.

The strength of our study is the large sample size compared to that in other studies and the control of confounding factors such as gestational age. Furthermore, our team comprises both obstetrics experts (MD, CB, and OM), who ensured the relevance of definitions of pathologies, and imaging experts with research engineers (BC and MB).

The main limitation of this study is the lack of clinical applicability of this technique. A promising recent study demonstrated the possibility to image the placental vascular tree in vivo [9]. Our results, combined with this technique, could provide to parents and obstetricians useful information during pregnancy. This trial is in accordance with the Standard Protocol Items: Recommendations for Interventional Trials checklist.

## Abbreviations

CHRU: Centre Hospitalier Régional Universitaire
CIC-IT: Centre D'investigation Clinical–Innovation Technological
CNIL: Commission Nationale de l'Informatique et des Libertés
e-CRF: electronic case report file
FVM: Fetal vascular malperfusion
GDPR: General Data Protection Regulation
IUGR: intrauterine growth restriction
MAPLE: MRI Angiography of Physiological and Pathological Pregnancy Placentas Ex vivo
Micro-CT: microcomputed tomography
MRI: magnetic resonance imaging
MVM: maternal vascular malperfusion
PE: preeclampsia

## Data Availability

All data generated during the project will be made freely available via Centre D'investigation Clinical–Innovation Technological (CIC-IT), Nancy. Data obtained from this study will be deposited at CIC-IT, Nancy, where they will be maintained for a minimum of 15 years. There are no security, licensing, or ethical issues related to the expected data, and all data used in the study will be generated directly as a result of the project, without any pre-existing data being used.
